# The Traditional Japanese Formula Keishibukuryogan Inhibits the Production of Inflammatory Cytokines by Dermal Endothelial Cells

**DOI:** 10.1155/2010/804298

**Published:** 2010-12-28

**Authors:** Yoko Yoshihisa, Megumi Furuichi, Mati Ur Rehman, Chieko Ueda, Teruhiko Makino, Tadamichi Shimizu

**Affiliations:** Department of Dermatology, Graduate School of Medicine and Pharmaceutical Sciences, University of Toyama, Sugitani, Toyama 930-0194, Japan

## Abstract

Keishibukuryogan (KBG) is one of the traditional herbal formulations widely administered to patients with blood stagnation for improving blood circulation; currently, it is the most frequently prescribed medicine in Japan. KBG has been reported to improve conjunctional microcirculation. The aim of this study was to evaluate the role of KBG and paeoniflorin, a bioactive compound of KBG, in inhibiting the production of inflammatory cytokines using human dermal microvessel endothelial cells (HDMECs). The authors observed that lipopolysaccharide (LPS; 1 *μ*g/mL) stimulated the secretion of proinflammatory cytokines in HDMECs. KBG treatment (10 mg/mL) significantly suppressed the mRNA levels of migration inhibitory factor (MIF), interleukin (IL)-6, IL-8, and tumor necrosis factor (TNF)-*α* in LPS-stimulated cultured HDMECs. Similarly, paeoniflorin significantly suppressed the mRNA levels of these cytokines in LPS-stimulated cultured HDMECs. ELISA showed that KBG and paeoniflorin suppressed the production of MIF, IL-6, IL-8, and TNF-*α* in LPS-stimulated HDMECs. Moreover, KBG and paeoniflorin decreased the expression of cyclooxygenase-2 and inducible nitric oxide synthase (iNOS) in these cells. These results suggest that KBG may be useful for improving microvascular inflammation in patients with skin diseases.

## 1. Introduction

Keishibukuryogan (KBG, Gui-zhi-fu-ling-wan in Chinese) is one of the Kampo medicines, and has been widely administered to patients with blood stagnation for improving blood circulation. KBG is now one of the most frequently used medicines in Japan. KBG has been used clinically to treat various diseases, including skin diseases. It was reported that KBG improves conjunctional microcirculation in patients with cerebrospinal vascular diseases [1], thus suggesting that it may have beneficial effects on hematological parameters such as blood viscosity and red blood cell deformability [2–4]. Moreover, Matsumoto et al. have explored a proteomic approach for the diagnosis of blood stasis in rheumatoid arthritis patients treated with KBG [5]. In addition, KBG is used to treat symptoms of peripheral ischemia such as cold extremities [6]. Furthermore, we recently reported that KBG is effective in patients with chronic pigmented purpura, a group of skin vascular disorders of unknown etiology [7]. 

KBG is composed of five medicinal plants, *Cinnamomum cassia* Blume (Cinnamomi cortex), *Paeonia lactiflora* Pallas (Paeoniae Radix), *Paeonia suffruticosa* Andrews (Moutan cortex), *Prunus persica* Batsch (Persicae semen) and *Poria cocos* Wolf (Hoelen) (Table 1) [8]. Paeoniae Radix and Moutan Cortex have many known active components in common, including paeoniflorin, paeonol, oxypaeoniflorin, benzoylpaeoniflorin, and palbinone [9]. Paeoniflorin is a characteristic main principal bioactive component of Paeoniae Radix, which included approximately 5.57% (w/w) paeoniflorin, and Moutan Cortex, which included approximately 3.96% (w/w) paeoniflorin [10]. Paeoniflorin has been reported to have many pharmacological effects, such as anti-inflammatory and antiallergic effects [11]. Recently, Zheng and Wei reported that the total glucosides present in the Moutan Cortex, which contain paeoniflorin as the principle bioactive component, inhibited primary and secondary inflammation in both collagen-induced arthritis and adjuvant-induced arthritis [12].

Up until now, topical and oral corticosteroids, oral bioflavonoids, ascorbic acid, griseofulvin, and cyclosporine have been suggested as treatments for chronic pigmented purpura [13, 14]. However, none of these treatments have proven to be satisfactory. Human dermal microvessel endothelial cells (HDMECs) are the prominent cells in dermal skin. HDMECs produce inflammatory cytokines, such as interleukin (IL)-6 and IL-8 when they are exposed to lipopolysaccharide (LPS). We therefore consider that examinations of the effects of KBG and paeoniflorin on HDMECs are important for the therapeutic studies of chronic pigmented purpura *in vitro*.

In this study, we aimed to evaluate the role of KBG and paeoniflorin, ininhibiting the production of inflammatory cytokines using HDMECs.

## 2. Materials and Methods

### 2.1. Materials

KBG (TJ-25) was obtained from Tsumura & Co. (Tokyo, Japan). KBG was suspended in CS-C complete medium containing 10% fetal calf serum and 1% penicillin and CSC growth factor (Cell Systems Inc, WA) and was rotated at 4°C overnight [11]. Then, the suspension was centrifuged, and the supernatant was filtered through a 0.45 *μ*m-pore membrane. The following materials were obtained from commercial sources: the Isogen RNA extraction kit was obtained from Nippon Gene (Tokyo, Japan); M-MLV reverse transcriptase was from GIBCO (Grand Island, NY); Taq DNA polymerase was from Perkin-Elmer (Norwalk, CO); LPS was purchased from Sigma (St. Louis); nylon membranes were from Schleicher & Schuell (Keene, NH); the anticyclooxygenase-2 (COX-2) polyclonal antibody (pAb) was purchased from Cell Signaling Technology, Inc. (Boston); antiinducible NOS (iNOS) pAb was purchased from Enzo Life Sciences International Inc. (NY); the anti-*β*-actin Ab was purchased from Santa Cruz Biotechnology Inc. (CA); Paeoniflorin was from LKT Laboratories, Inc.; MIF, IL-6, and IL-8 ELISA kits were obtained from R&D Systems (Minneapolis); and the Western blot detection system was obtained from Cell Signaling Technology (Beverly, MA). All other reagents were of analytical grade.

### 2.2. Cell Stimulation

Primary human dermal endothelial cells (HDMECs) were obtained from Cell Systems Inc (WA). HDMECs were cultivated in conditioned endothelial culture medium (CS-C complete medium) containing 10% fetal calf serum and 1% penicillin and CSC growth factor at 37°C in a 5% CO_2_ atmosphere. Cell viability before treatment was always over 95% as evaluated by Trypan blue dye exclusion test. On the day of the experiment, cells were collected and suspended in fresh culture medium at a concentration of 1 × 10^6^ cells/mL. The cells were divided into 4 groups: a control group, a group receiving 10 mg/mL of KBG or 100 *μ*g/mL of paeoniflorin as previously described [9, 15], a group treated with 1 *μ*g/mL of LPS, and another group treated with the combination. The cells were treated with the various agents, and analyses were performed as described for each procedure.

### 2.3. MTT Assay

The number of cells was measured by an MTT assay. In brief, the media were removed by aspiration, and then the cells were treated with 0.5 mg/mL dimethylthiazol-2-yl-2,5-diphenyltetrazolium bromide (MTT, Sigma) in culture medium for 4 hours at 37°C. After being washed with PBS once, isopropanol/0.04 M HCl was added, and OD570 was measured, and the value of blank was subtracted.

### 2.4. Reverse Transcription-PCR Analysis

Total RNA was extracted from each mouse skin specimen. RNA reverse transcription was performed with M-MLV reverse transcriptase using random hexamer primers, and subsequent amplification was done using Taq DNA polymerase. PCR was carried out for 30 cycles with denaturation at 94°C for 30 seconds, annealing from 52 to 64°C for 1 minute and extension at 72°C for 30 seconds using a thermal cycler (PE Applied Biosystems Gene Amp PCR system 9700). The human MIF primers used were 5^'^-ATGCCGATGTTCATCGTAAAC-3^'^ (forward) and 5^'^-GGCGAAGGTGGAGTTGTTCCA-3^'^ (reverse). The IL-6 primers used were 5^'^-GATGCAATAACCACCCCTGACCC-3^'^ (forward) and 5^'^-CAATCTGAGGTGCCCATGCTAC-3^'^ (reverse). The IL-8 primers used were 5^'^-CATGACTTCCAAGCTGGCCGTG-3^'^ (forward), 5^'^-CCACTCTCAATCACTCTCAGTTC-3^'^ (reverse) [16]. The TNF-*α* primers used were 5^'^-ACACCGTCAGCCGATTTGC-3^'^ (forward) and 5^'^-CCCTGAGCCATAATCCCCTT-3^'^ (reverse). GAPDH was used as a positive control. The primers used were 5^'^-ACCCAGAAGACTGTGGAT-3^'^ (forward) and 5^'^-TCGTTGAGGGCAATGCCA-3^'^ (reverse). After PCR, the amplified products were analyzed using 2% agarose gel electrophoresis.

### 2.5. Cytokine Release Measurements

Supernatants were collected after 24 h of incubation with LPS (1 *μ*g/mL) and/or KBG (10 mg/mL) and paeoniflorin (100 *μ*g/mL). MIF, IL-6, and IL-8 were assayed by ELISA, according to the manufacturer's instructions. The absorbance was measured with a microplate reader (Labsystems Multiskan Bichromatic).

### 2.6. Western Blot Analysis

Cells were collected and washed with cold PBS. The cells were lysed at a density of 1×10^6^ cells/50 *μ*L of RIPA buffer (1 M Tris-HCA, 5 M NaCl, 1% Nonidet P-40 (v/v), 1% sodium deoxycholate, 0.05% SDS, 1 mM phenylmethyl sulfonyl fluoride) for 20 minutes. After brief sonication, the lysates were centrifuged at 12,000 rpm for 10 minutes at 4°C, and the protein content in the supernatants was measured using a bio-rad protein assay kit (Bio-Rad, Hercles, CA). The protein lysates were denatured at 96°C for 5 min after mixing with 5 *μ*L of sodium dodecylsulfate (SDS) loading buffer, applied on an SDS polyacrylamide gel for electrophoresis, and transferred to nitrocellulose membranes. Western blot analysis was carried out to detect the expression levels of COX-2 and iNOS using specific antibodies [17]. Band signals were visualized on X-ray film using chemiluminescence ECL detection reagents (Amersham Biosciences, Buckinghamshire, UK). The relative amounts of proteins associated with specific antibodies were normalized according to the intensities of *β*-actin.

### 2.7. Statistical Analysis

The values are expressed as the means ± SD of the respective test or control group. Statistically significant differences in the stimulation with LPS in the groups treated with KBG or paeoniflorin were evaluated by the nonparametric Mann-Whitney *U* test. *P*-values of <.05 were considered to be statistically significant.

## 3. Results

### 3.1. The Effects of KBG on LPS-Stimulated Induction of Cytokines in HDMECs

**Figure 1:**
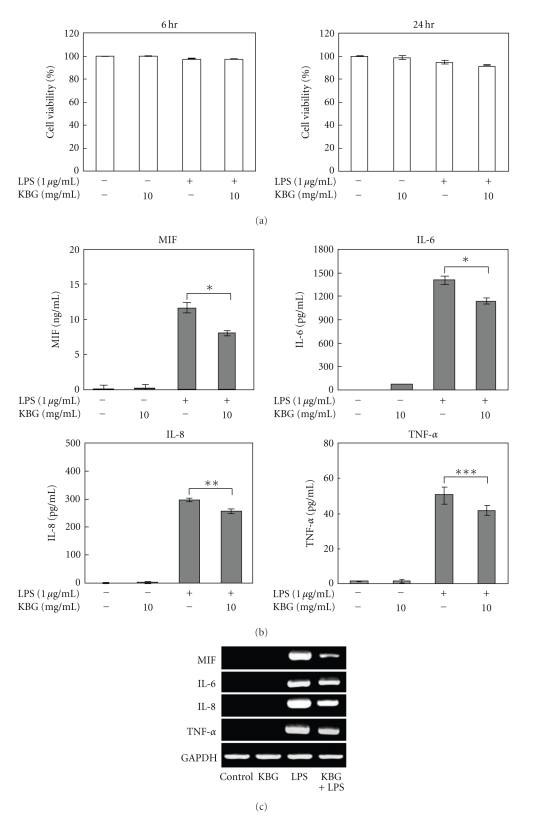
Effect of KBG on LPS-stimulated induction of cytokines in HDMECs. (a) HDMEC cells were treated with 1 *μ*g/mL LPS and/or KBG (10 mg/mL) for 6 or 24 h and were subjected to the MTT assay. (b) HDMEC cells were treated with 1 *μ*g/mL LPS and/or 10 mg/mL of KBG for 24 h. The MIF, IL-6, IL-8, and TNF-*α* content of cultured supernatants was analyzed by ELISA. Data are presented as the means ± S.D (*n* = 5). *P < .001, **P < .005, ***P < .05. (c) The cells were treated with 1 *μ*g/mL LPS and/or 10 mg/mL of KBG for 6 h. Total RNA was isolated, and the mRNA expression levels of MIF, IL-6, IL-8, and TNF-*α* were detected by RT-PCR. (These experiments were repeated three times with similar results).

LPS plays a fundamental role in the pathogenesis of a number of inflammatory diseases by inducing a distinctive pattern of inflammatory cytokine release. We used LPS to produce inflammatory conditions in HDMECs. The cells were incubated with or without 1 *μ*g/mL of LPS or 10 mg/mL of KBG for 6 or 24 h, and the cell viability was assessed. None of the treatments elicited cytoxicity in the cells at the tested concentrations and incubation times (Figure 1(a)). We examined the anti-inflammatory effects of KBG on the induction of inflammatory cytokines by LPS stimulation. LPS-induced protein levels of MIF, IL-6, IL-8, and TNF-*α* were significantly inhibited by KBG (Figure 1(b)). Treatment of the cells with 1 *μ*g/mL of LPS increased in mRNA levels of these cytokines from 6 h after stimulation, but these mRNA levels were decreased by KBG (Figure 1(c)).

### 3.2. Effect of Paeoniflorin on LPS-Stimulated Induction of Cytokines in HDMECs

**Figure 2:**
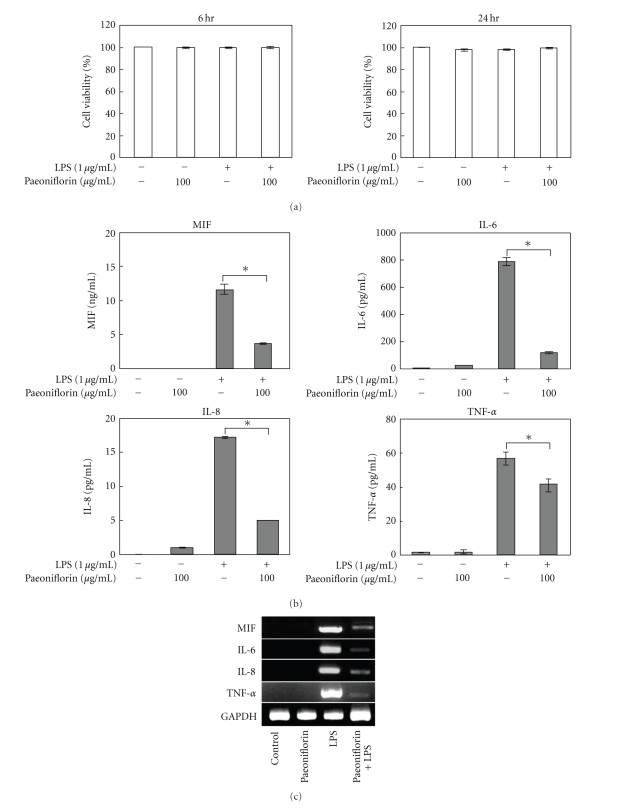
Effect of paeoniflorin on LPS-stimulated induction of cytokines in HDMECs. (a) HDMEC cells that were treated with LPS (1 *μ*g/mL) and/or paeoniflorin (100 *μ*g/mL) for 6 or 24 h and were subjected to the MTT assay. (b) HDMEC cells were treated with 1 *μ*g/mL LPS and/or 100 *μ*g/mL of paeoniflorin for 24 h. The MIF, IL-6, IL-8, and TNF-*α* protein content of cultured supernatants was analyzed by ELISA. Data are presented as the means ± S.D (*n* = 5). *P < .0005. (c) The cells were treated with 1 *μ*g/mL LPS and/or 100 *μ*g/mL of paeoniflorin for 6 h. Total RNA was isolated, and the mRNA expression levels of MIF, IL-6, IL-8, and TNF-*α* were detected by RT-PCR. (These experiments were repeated three times with similar results).

The cells were incubated with or without 1 *μ*g/mL of LPS or 100 *μ*g/mL of paeoniflorin for 6 or 24 h, and the cell viability was assessed.  Figure 2(a) reveals that the incubation with paeoniflorin did not elicit toxicity in cells at the tested concentration at either incubation time. LPS-induced protein levels of MIF, IL-6, IL-8, and TNF-*α* were significantly inhibited by paeoniflorin, and the inhibitory effect was stronger than that of KBG (Figure 2(b)). Moreover, to examine the effects of paeoniflorin on the mRNA levels of inflammatory cytokines, cells were treated with LPS for 6 h in the presence of 100 *μ*g/mL of paeoniflorin. The LPS-induced MIF, IL-6, IL-8, and TNF-*α* mRNA levels were decreased by paeoniflorin (Figure 2(c)).

### 3.3. Inhibition of COX-2 and iNOS Protein Expression by Paeoniflorin

**Figure 3:**
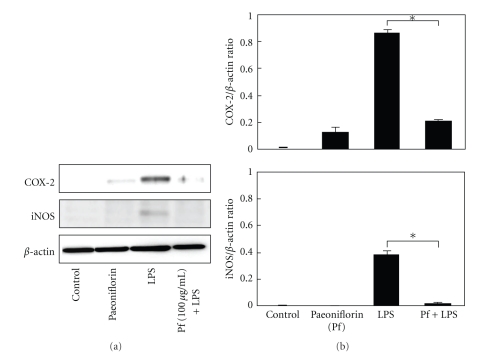
Effect of paeoniflorin COX-2 and iNOS protein expression. HDMEC cells were treated with 1 *μ*g/mL LPS and/or 100 *μ*g/mL of paeoniflorin for 24 h. COX-2 and iNOS protein expression in the cells was detected by a Western blot analysis, using *β*-actin as the internal control. The results of densitometric analysis, normalized with respect to *β*-actin using a bioimaging analyzer, are presented. Data are presented as the means ± S.D (*n* = 5). *P < .0001. (This experiment was repeated three times with similar results).

Next, we examined whether paeoniflorin could inhibit the production of COX-2 and iNOS. Western blot analyses revealed that the expression of COX-2 and iNOS was increased by LPS stimulation in HDMECs whereas the treatment with paeoniflorin resulted in a decrease in COX-2 and iNOS expression (Figure 3).

## 4. Discussion

Among traditional Japanese medicinal compounds, Kampo formulas originated from ancient Chinese medicine, and are currently recognized and reimbursed by the Health Ministry in Japan for the treatment of a wide variety of conditions. The Kampo formula KBG is frequently used in traditional Japanese and Chinese medicine to treat several symptoms, including skin diseases. The preparation has demonstrated anti-inflammatory and free-radical scavenging effects. 

In the present study, we have demonstrated that KBG treatment significantly suppressed the protein and mRNA levels of MIF, IL-6, IL-8, and TNF-*α* in LPS-stimulated cultured HDMECs. Chronic pigmented purpura is a disease complex characterized by capillaritis and lymphocytic capillary damage in the papillary dermis. Several lines of evidence suggest the involvement of humoral or cellular immunity, as well as hemostasis, in the pathogenesis of this disease [18]. These patients are sometimes resistant to standard treatments, such as topical corticosteroids. Indeed, our previous data support the effectiveness of KBG, which led to a significant improvement in patients with chronic pigmented purpura [7]. It is reported that KBG can prevent the progression of atherosclerosis [19], and preserve vascular endothelial function in cholesterol-fed rabbits [19] and in hypertensive rats [20]. Furthermore, in diabetic rats, it was demonstrated to have protective effects against vascular injury [21], and to delay the development of diabetic kidney disease [22, 23]. Moreover, KBG has a favorable effect on impaired glucose metabolism in type 2 diabetes by improving glucose intolerance, and it has been suggested that some of this effect is derived from the reduction of TNF-*α* content in skeletal muscle [24]. Accordingly, KBG may exert beneficial effects that result in the inhibition of inflammatory cytokines in HDMECs. It was recently reported that MIF induces the endothelial expression of IL-8, thus resulting in leukocyte recruitment [25]. Since MIF is an initiator of other proinflammatory cytokines such as IL-6, IL-8, and TNF-*α*, and regulates the induction of these cytokines [26, 27], we hypothesized that KBG may inhibit MIF-regulated inflammatory mediators.

We also demonstrated that paeoniflorin, a bioactive compound contained in KBG, suppressed the mRNA levels of these inflammatory cytokines in LPS-stimulated cultured HDMECs, similar to the effects of KBG. It is known that paeoniflorin has anti-inflammatory and antiallergic activities [28, 29]. In addition, paeoniflorin has reportedly exhibited immunoregulatory effects [30, 31], analgesic effects [32], neuromuscular blocking [33, 34], cognition enhancement [35], and steroid protein binding inhibition [36]. Our study revealed that paeoniflorin decreased the expression of COX-2 and iNOS in HDMECs. Many studies have demonstrated that overproduction of nitric oxide and inflammatory prostaglandins such as PGE2, mainly via iNOS and COX-2, respectively, plays an important pathophysiological role in the development of inflammation [37]. Kim and Ha reported that paeoniflorin treatment significantly attenuated LPS-induced NO and PGE2 production [38]. Moreover, the extract of KBG has been shown to have COX-2 and inducible iNOS inhibitory activities [39]. In this context, we suggest that KBG and paeoniflorin induce their anti-inflammatory effects by inhibiting both proinflammatory cytokines and inhibiting the cascade of the overproduction of COX-2 and iNOS in HDMECs.

## 5. Conclusion

Taken altogether, these findings suggest that KBG may be useful to improve microvascular inflammation in patients with skin diseases such as chronic pigmented purpura.

## Figures and Tables

**Table 1:** Components of keishibukuryogan (KBG).

Japanese name	Scientific name	Botanical name	Ratio (g)
Keihi	Cinnamomi cortex	*Cinnamomum cassia* Blume	1
Syakuyaku	Paeoniae radix	*Paeonia lactiflora* Pallas	1
Tounin	Persicae semen	*Prunus persica* Batsch	1
Bukuryou	Hoelen	*Poria cocos* Wolf	1
Botanpi	Moutan cortex	*Paeonia suffruticosa* Andrews	1

## Nonstandard Abbreviations:

ELISA: Enzyme-linked immunosorbent assay
HDMECs: Human dermal microvessel endothelial cells
KBG: Keishibukuryogan
IL: Interleukin
LPS: Lipopolysaccharide
MIF: Migration inhibitory factor
TNF: Tumor necrosis factor.
